# Promising abscopal effect of combination therapy with thermal tumour ablation and intratumoural OK-432 injection in the rat osteosarcoma model

**DOI:** 10.1038/s41598-020-66934-6

**Published:** 2020-06-15

**Authors:** Tadashi Iwai, Naoto Oebisu, Manabu Hoshi, Kumi Orita, Akira Yamamoto, Shinichi Hamamoto, Ken Kageyama, Hiroaki Nakamura

**Affiliations:** 1 Department of Orthopedic Surgery, Osaka City University Graduate School of Medicine, 1-4-3 Asahi-Machi, Abeno-Ku, Osaka, 545-8585 Japan; 2 Department of Radiology, Osaka City University Graduate School of Medicine, 1-4-3 Asahi-Machi, Abeno-Ku, Osaka, 545-8585 Japan

**Keywords:** Cancer, Oncology

## Abstract

Treatment options for metastatic osteosarcoma are limited. The present study aimed to evaluate whether radiofrequency ablation (RFA) combined with intratumoural OK-432 injection induces systemic anti-tumour immunity in rat osteosarcoma model. Eighty of 145 rats were assigned to four groups to evaluate overall survival and tumour size: control (no treatment), RFA-only, OK-432, and RFA-OK-432. The remaining 65 were assigned for histological examination. Maximum diameters of tibial and lung tumours were determined. Tumour samples were histologically examined using haematoxylin-eosin and immunohistochemical staining. Overall survival was significantly prolonged in the RFA-OK-432 group compared to the RFA-only and OK-432 groups. Only rats in the RFA-OK-432 group exhibited significant decreases in maximum tumour diameter after treatment. Ki-67-positive tumour cells in the RFA-OK-432 group were significantly stained negative on immunohistochemical analysis as opposed to those in the RFA-only and OK-432 groups. The number of CD11c+, OX-62+, CD4+, and CD8 + cells significantly increased in the RFA-OK-432 group compared to the RFA-only group. RFA with intratumoural OK-432 injection resulted in distant tumour suppression, prolonged survival, and increased dendritic cells, cytotoxic T cells, IFN-γ, and TNF-α, whereas RFA or OK-432 alone did not produce this effect. This combination may induce an abscopal effect in human osteosarcoma.

## Introduction

The prognosis of non-metastatic osteosarcoma has significantly improved after the mid-1970s with the development of multi-agent chemotherapy. However, for patients with unresectable osteosarcoma with multiple metastases, no effective treatment has been established. Systemic chemotherapy is the only treatment option but is ineffective in most such cases. Thus, the 5-year survival rate associated with osteosarcoma with metastasis is less than 20%^1^.

Assessment of the effect of radiofrequency ablation (RFA) has mainly focused on its direct local effect, i.e., the maximum extent to which target tumours can be ablated. Recently, an indirect systemic effect (the so-called abscopal effect—a phenomenon of regression at a site distant from the primary site of RFA), has also gained attention^2^. Among patients with advanced kidney and liver cancer, RFA has been performed more frequently to treat both primary and advanced cancers^3,4^. Moreover, in patients with advanced bone and soft tissue sarcoma, RFA has proven useful for lung metastases and can be expected to have an outcome similar to that of metastasectomy, prohibiting further tumour progression and increasing overall survival^5^.

OK-432 is a freeze-dried product prepared by incubating the low-virulence Su strain of group A *Streptococcus pyogenes* with penicillin^6^. Previous reports have demonstrated aspects of its efficacy, including prolonged survival in patients with postoperative stomach cancer or primary lung cancer in combination with chemotherapy^7,8^; reduction of malignant pleural effusion in advanced gastrointestinal cancer or lung cancer^9^, head and neck cancer, and thyroid cancer that are unresponsive to systemic chemotherapy^10,11^; and lymphangioma^12^. Furthermore, Nishida *et al*. demonstrated that combination therapy with re-implantation of tumour tissue after cryotherapy and OK-432 injection synergistically activated the immune response and inhibited metastatic tumour growth in a murine osteosarcoma model^13^. Therefore, we hypothesised that a combination therapy involving RFA and OK-432 injection could also synergistically induce systemic anti-tumour immunity.

The purpose of the present study was to evaluate the efficacy of RFA and assess whether a combination therapy comprising RFA and intratumoural OK-432 injection induces systemic anti-tumour immunity in a rat advanced osteosarcoma model established using the osteosarcoma cell line (UMR106).

## Results

### Tumour implantation

After implantation of UMR106 cells in 160 Sprague-Dawley rats, the tumour cells failed to colonise either the left tibias or right lungs of 15 rats (9.4%). The 145 rats (90.6%) that showed tumour establishment were used for our study.

### Survival

All 35 rats in the RFA-only group successfully underwent RFA of left tibial tumours. All 35 rats in the RFA-OK-432 group also underwent RFA. There were no complications with the treatment.

The mean period of survival was 28.4 (range, 11–51) days in the control group, 40.0 (range, 12–51) days in the RFA-only group, 38.4 (range, 10–51) days in the OK-432 group, and 47.3 (range, 15–51) days in the RFA-OK-432 group (Fig. 2). Rats in the RFA-OK-432 group had significantly longer survival periods than did those in the control, RFA-only, and OK-432 groups (*P* = 0.002, 0.047, and 0.046, respectively). However, there were no significant differences between the RFA-only and control groups (P = 0.084).

### Primary tumour growth

The mean maximum diameters of primary tumours before treatment were 13.1 ± 2.2 (range, 9–16) mm, 13.0 ± 2.5 (range, 8–17) mm, 13.0 ± 2.0 (range, 10–17), and 14.5 ± 1.8 (range, 11–18) mm in the control, RFA-only, OK-432, and RFA-OK-432 groups, respectively (Fig. 3). There were neither significant differences among the four groups before treatment nor on days 16 or 30. Rats in the RFA-OK-432 group had significantly smaller maximum diameters of primary tumours on days 51 than did those in the control group (*P* = 0.008). However, the maximum diameters of primary tumours among rats in the RFA-only group on day 51 were not significantly smaller than those of the control group (*P* = 0.26). There were also no significant differences between the RFA-OK-432 and RFA-only groups on days 16, 30, and 51 (*P* = 1.00, 0.84, and 0.39, respectively) (Fig. 3).

### Distant tumour growth

The mean diameters of distant tumours before treatment were 14.3 ± 3.3 (range, 8–21) mm, 12.5 ± 6.5 (range, 3–24.5) mm, 11.7 ± 5.3 (range, 5–24) mm, and 11.7 ± 6.6 (range, 4.5–32) mm in the control, RFA-only, OK-432, and RFA-OK-432 groups, respectively (Fig. 3). There were no significant differences among the four groups before treatment. After treatment, the tumour diameters of rats in the RFA-only, OK-432, and RFA-OK-432 groups were smaller than those of rats in the control group.

On days 16, 30, and 51, rats in the RFA-OK-432 group had significantly smaller tumours than did those in the control group (*P* = 0.03, 0.007, and 0.004, respectively). However, the maximum diameters of distant tumours among rats in the RFA-only and OK-432 groups on day 51 were not significantly smaller than those of rats in the control group (*P* = 0.24 and 0.07 respectively). Moreover, there were no significant differences in tumour diameter between the RFA-OK-432 and RFA-only groups on days 16, 30, and 51 (*P* = 0.70, 0.56, and 0.25, respectively) (Fig. 3).

On day 51, there were significantly smaller changes in the diameters of the right lung tumours among rats in the RFA-OK-432 group (*P* < 0.001) while rats in the RFA-only and OK-432 groups showed no remarkable change. The maximum diameters of the right lung tumours among rats in the control group were significantly larger (*P* < 0.001).

### Findings on haematoxylin-eosin examination and immunohistochemistry for Ki-67-positive tumour cells in primary and distant tumours

The findings of examination via haematoxylin-eosin staining and immunohistochemical staining for Ki-67 positivity in primary and distant tumours on days 16, 30, and 51 are shown in Figs. 4–6. Concerning the primary tumours, the number of Ki-67-positive tumour cells in the RFA-OK-432 group was significantly lower than that in the control, RFA-only, and OK-432 groups (all *P* < 0.05). Furthermore, there were significant differences in the number of Ki-67-positive tumour cells between the RFA-only and control groups (all *P* < 0.05) (Fig. 7).

Concerning distant tumours, the number of Ki-67-positive tumour cells in the RFA-OK-432 group was significantly lower than in the control group (all *P* < 0.001). The number of Ki-67-positive tumour cells among rats in the RFA-OK-432 group was also significantly lower than that among rats in the RFA-only and OK-432 groups (all *P* < 0.05). Furthermore, there were significant differences in the number of Ki-67-positive tumour cells between the RFA-only and control groups on day 51 (*P* < 0.001) (Fig. 7).

### CD11c+, OX-62+, CD4+, and CD8 + cells in primary tumours

The results of the immunohistochemical examination of CD11c+, OX-62+, CD4+, and CD8 + cells in the primary tumours on days 16, 30, and 51 are shown in Figs. 4–6. After treatment, the number of CD11c + cells in primary tumours among rats in the RFA-OK-432 group was significantly higher than that among rats in the control, RFA-only, and OK-432 groups (all *P* < 0.01). The number of CD11c + cells in primary tumours among rats in the RFA-only and OK-432 groups was also significantly higher than that among rats in the control group (all *P* < 0.01). Moreover, the number of OX-62+ cells in primary tumours among rats in the RFA-OK-432 group was also significantly higher than that among rats in the control and OK-432 groups (all *P* < 0.05). The number of OX-62+ cells in primary tumours among rats in the RFA-only and OK-432 groups was significantly higher than that among rats in the control group (all *P* < 0.001) (Fig. 7).

The number of CD8 + cells in primary tumours among rats in the RFA-OK-432 group was significantly higher than that among rats in the control group (all *P* < 0.001). The number of CD4 + cells in primary tumours among rats in the RFA-OK-432 group was also significantly higher than that among rats in the control, RFA-only, and OK-432 groups (all *P* < 0.05). Moreover, the number of CD8 + cells in primary tumours among rats in the RFA-only and OK-432 groups was significantly higher than that among rats in the control group on days 30 and 51 (all *P* < 0.001). The number of CD4 + cells in primary tumours among rats in the RFA-only and OK-432 groups was also significantly higher than that among rats in the control group (all *P* < 0.01) (Fig. 7).

### CD11c+, OX-62+, CD4+, and CD8 + cells in distant tumours

The results of the immunohistochemical examination of CD11c+, OX-62+, CD4+, and CD8 + cells in the distant tumours on days 16, 30, and 51 are shown in Figs. 4–6. After treatment, the number of CD11c + cells in distant tumours among rats in the RFA-OK-432 group was significantly higher than that among rats in the control, RFA-only, and OK-432 groups (all *P* < 0.001). The number of CD11c + cells in distant tumours among rats in the OK-432 group was also significantly higher than that among rats in the control group (all *P* < 0.01). Moreover, the number of OX-62+ cells in distant tumours among rats in the RFA-OK-432 group was also significantly higher than that among rats in the control, RFA-only, and OK-432 groups (all *P* < 0.01). The number of OX-62+ cells in distant tumours among rats in the RFA-only groups was significantly higher than that among rats in the control group (all *P* < 0.05). The number of OX-62+ cells in distant tumours among rats in the OK-432 groups was significantly higher than that among rats in the control group on days 16 and 30 (all *P* < 0.05) (Fig. 7).

The number of CD8 + cells in distant tumours among rats in the RFA-OK-432 group was significantly higher than that among rats in the control and RFA-only groups (all *P* < 0.05). The number of CD4 + cells in distant tumours among rats in the RFA-OK-432 group was also significantly higher than that among rats in the control, RFA-only, and OK-432 groups (all *P* < 0.05). Moreover, the number of CD8 + cells in distant tumours among rats in the RFA-only and OK-432 groups was significantly higher than that among rats in the control group on days 30 and 51 (all *P* < 0.05). The number of CD4 + cells in distant tumours among rats in the RFA-only and OK-432 groups was also significantly higher than that among rats in the control group on days 30 and 51 (all *P* < 0.001) (Fig. 7).

### Cytokine levels in the blood

The amount of IFN-γ and TNF-α was also examined on day 30 using ELISA. The amount of IFN-γ and TNF-α in the blood among rats in the RFA-OK-432 group was significantly higher than that among rats in the control, RFA-only, and OK-432 groups (all *P* < 0.05). However, there was no significant difference in the amount of IFN-γ and TNF-α between the RFA-only and control groups (*P* = 0.73 and 0.88, respectively). Furthermore, there was no significant difference in the amount of IFN-γ and TNF-α between the OK-432 and control groups (*P* = 0.49 and 0.99, respectively) (Fig. 8).

## Discussion

Osteosarcoma is the most frequently diagnosed primary malignant bone tumour, particularly among children and young people globally^16^. In Japan, the annual total number of newly diagnosed cases of human osteosarcoma is low; the incidence is 1–1.5 per 1,000,000 population, and more than 70% of osteosarcoma patients are younger than 40 years^17^. Osteosarcomas originate more frequently in the metaphyseal region of tubular long bones with 50% and 20% occurring in the femur and tibia, respectively^17^. The treatment of primary osteosarcoma without metastases generally consists of surgery and preoperative and postoperative chemotherapy with doxorubicin, cisplatin, and high-dose methotrexate, which generally achieves 5-year overall survival rates of 60–80%^18^. However, patients with metastatic or unresectable osteosarcomas have limited treatment options and low progression-free survival and overall survival rates^19^. Therefore, new strategies for anti-tumour therapy are required.

RFA is a commonly used treatment modality that directly heats the tumour tissue, and the temperature can reach 60–100 °C, which results in coagulation necrosis of tumour tissue. It also requires the insertion of one or more radiofrequency applicators into a target lesion using image guidance, generally ultrasound or computed tomography. In addition to causing irreversible thermal damage to tumour cells, RFA stimulates the whole body to produce specific immunity. Recently, this immune response has been thought to have the potential to treat metastatic cancer effectively^20^. In particular, it has been demonstrated that RFA induces an anti-tumour response, which is referred to as the abscopal effect. The abscopal effect has been largely reported in radiotherapy studies and is enhanced with combined immunotherapies^20^.

The essential process of tumour immunity can be explained by the cancer immunity cycle^21^. In phase 1 of this cycle, dendritic cells (DCs) capture tumour antigens produced in dead tumour cells. DCs then present the antigens to T lymphocytes and move to the lymph node where they become mature DCs (phase 2). Mature DCs stimulate cytotoxic T lymphocytes (CTLs), especially CD4+ and CD8+ T cells (phase 3). Stimulated tumour-specific CTLs move to the tumour (phase 4). Tumour-specific CTLs invade the tumour (phase 5). Tumour-specific CTLs identify tumour cells through major histocompatibility complex class I molecules (phase 6). Tumour-specific CTLs destroy cancer cells, and tumour antigens are released (phase 7). The cancer immunity cycle is repeated over and over again. However, various factors can regulate this cancer immunity cycle, both positively and negatively. For example, tumour antigens released from destroyed cancer cells, heat shock protein and high morbidity group box-1 produced by heat coagulation, and both pro-inflammatory and anti-inflammatory cytokines are released or expressed after RFA. Therefore, RFA can induce an anti-tumour immune response. However, anti-inflammatory cytokines regulate the cancer immunity cycle negatively. Several reports have revealed that RFA combined with immunomodulation can regulate the cancer immunity cycle positively.

OK-432 was approved by the Ministry of Health and Welfare in Japan as an anti-cancer agent in 1975. It is administered as a systemic injection (intramuscularly, subcutaneously, or intradermally) or as an intratumoural or intraserosal injection for specific cancers. A previous report indicated that OK-432 promoted the activation of DCs in phase 2 of the cancer immunity cycle^22^.

DCs are specialised cells that play an important role in the induction of immunity. Tumour antigen-loaded mature DCs are currently being exploited in the formulation of cancer vaccines in various clinical studies. Brok *et al*. reported that RFA induced anti-tumour immunity by both increasing and maturing DCs in a mouse melanoma model^23^. Nakagawa *et al*. combined RFA and mature DC injection, which was stimulated by OK-432, and found a significant tumour growth inhibition^24^. Hamamoto *et al*. also reported that combining RFA with a local injection of OK-432 inhibited the growth of distant tumours and enhanced overall survival^25^. Thus, there have been various reports about the systemic anti-cancer effect of the combination of RFA treatment with local OK-432 injection.

The present study showed that combining RFA with intratumoural OK-432 injection could enhance the anti-tumour efficacy of RFA or OK-432 on its own in the rat osteosarcoma model. Our results also showed that RFA alone or OK-432 alone could result in an insufficient immunological effect. A combination of RFA and intratumoural OK-432 injection significantly prolonged the overall survival and induced distant tumour regression. Histopathologically, the number of Ki-67-positive tumour cells in both primary and distant tumours was significantly lower, and the number of not only CD11c + and OX-62+ DCs but CD4+ and CD8+ T cells in both primary and distant tumours was significantly higher after RFA in combination with intratumoural OK-432 injection. As a result, we suspected that the reduction in the number of tumour cells was induced by increasing the number of not only CD11c+ and OX-62+ DCs but CD4+ and CD8+ T cells. To our knowledge, this is the first study to show that the treatment of primary osteosarcoma via RFA could induce systemic anti-tumour effects.

The proliferation marker Ki-67, assessed immunohistochemically, was previously shown to be a strong prognostic factor, particularly in breast cancer^26^ and Ewing’s sarcoma^27^. Recently, it was reported that changes in Ki-67-positive cells among osteosarcoma patients before and after treatment had a poor prognostic value^28^. In the present study, the number of Ki-67-positive tumour cells among rats in the RFA-OK-432 was significantly lower than that among rats in the control group, and the survival duration of rats in the RFA-OK-432 group was significantly longer than that among rats in the control group. There was no significant difference in the size of both primary and distant tumours between the RFA-OK-432 and RFA-only groups at each time point (Fig. 3). However, the number of Ki-67-positive tumour cells of both primary and distant tumours in the RFA-OK-432 group was significantly lower than that in the RFA-only group at each time point (Fig. 7). In fact, rats in the RFA-OK-432 group had significantly longer survival periods than did those in RFA-only group (Fig. 2). Based on these results, the number of Ki-67-positive tumour cells may also be a significant prognostic factor.

Various studies have confirmed the association of high densities of tumour-infiltrating not only CD11c+ or/and OX-62+ DCs but CD4+ or/and CD8+ T cells with a better overall survival^29–32^. In the present study, the number of distant tumour-infiltrating not only CD11c+ and OX-62+ DCs but CD4+ and CD8+ T cells in the RFA-OK-432 group was higher than that in the control or RFA-only group (Fig. 7). The number of distant tumour-infiltrating not only CD11c+ and OX-62+ DCs but CD4+ T cells in the RFA-OK-432 group was also higher than that in the OK-432 group (Fig. 7). Moreover, the number of tumour-infiltrating CD4+ and CD8+ T cells seemed to be lower in most rats that died soon after treatment.

Tumour-infiltrating CD4+ and CD8+ T cells have been reported to be associated with increased IFN-γ and TNF-α synthesis inducing tumour-specific T cell response (33). In the present study, the amount of IFN-γ and TNF-α in the RFA-OK-432 group was also higher than that in the control, RFA-only, and OK-432 groups (all *P* < 0.05).

The present study has several limitations. First, we defined the left tibial tumour as the primary tumour and the right lung tumour as the metastatic tumour in the rat osteosarcoma model, which appears to be non-physiological. However, some studies that used a mouse colon cancer model also defined the primary tumour as the treated tumour on one flank, and the metastatic tumour as the untreated tumour on the contralateral flank^24,33^. An ideal model would be one in which the metastatic lung tumour was defined as the left lower leg tumour of the rats. Second, we did not perform quantitative assessments via flow cytometry of tumour-infiltrating lymphocytes. However, we performed qualitative and semi-quantitative assessments via both haematoxylin-eosin and immunohistochemical examination of tumours. Moreover, we quantitatively assessed immunohistochemistry slides using ImageJ. Furthermore, the optimal dose of intratumoural OK-432 injection is yet to be validated. We used 0.075 KE (0.5 KE/kg) in the present study based on a previous report^34^. As a result, we indicated that the efficacy of OK-432 was approximately equivalent to that of RFA, while various authors had reported that the efficacy of OK-432 was slightly weaker than that of RFA^25,35^. Besides, the extent of the immune response in rats differs from that in humans; therefore, it is unclear whether the same results would be obtained in humans. However, previous studies have reported that RFA stimulates anti-tumour immunity in colon cancer with liver metastases in both rats and humans^36,37^. A combination of RFA and intratumoural injection of OK-432 may also be capable of stimulating anti-tumour immunity in human metastatic osteosarcoma.

In conclusion, RFA for left tibial osteosarcoma in combination with intratumoural OK-432 injection led to prolonged survival and inhibition of distant untreated lung osteosarcoma growth in the rat osteosarcoma model. These results indicate that this combination therapy may also induce a promising abscopal effect in human metastatic osteosarcoma.

## Methods

**Figure 1:**
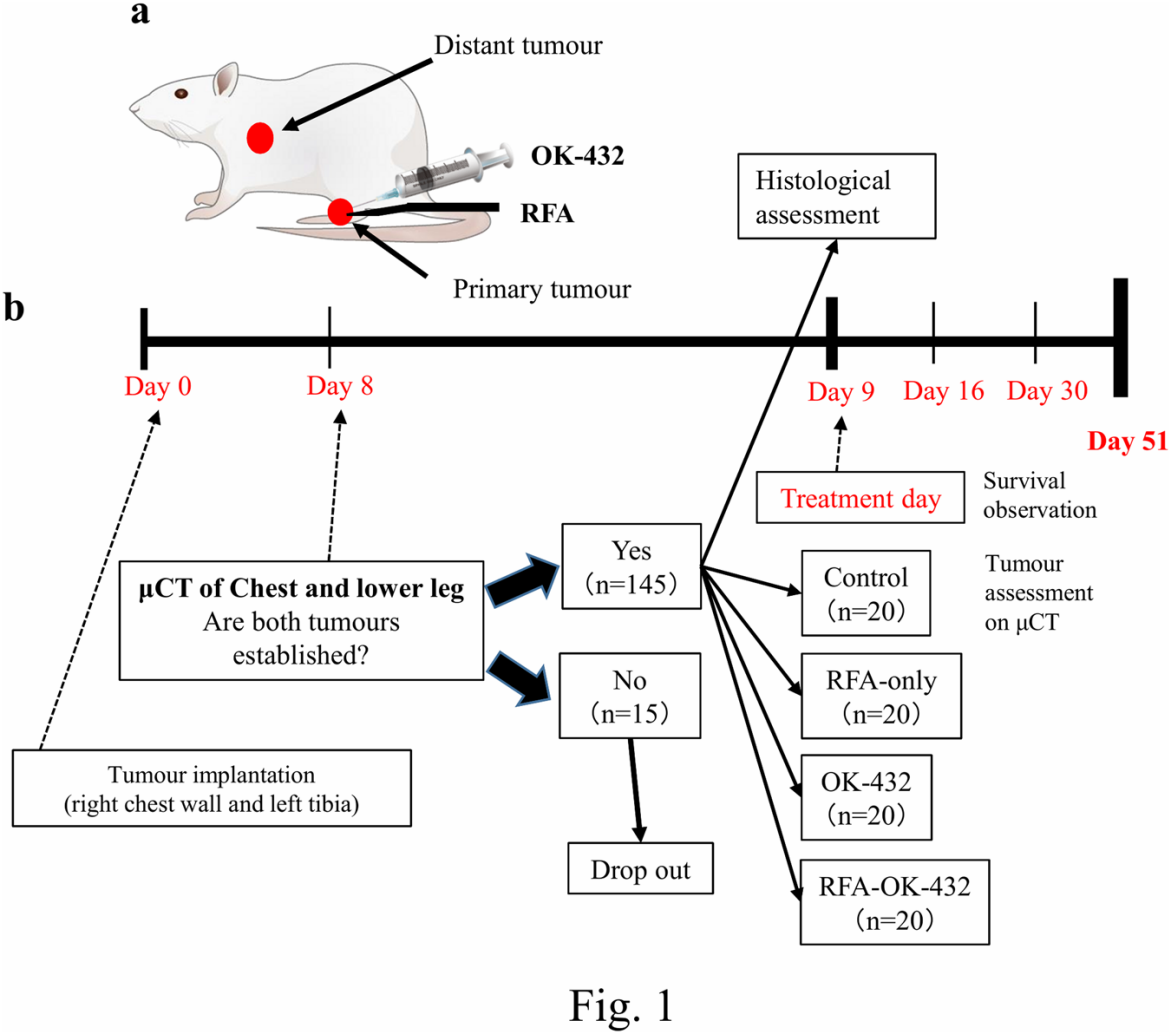
RFA and intratumoural OK-432 injections were administered into the left tibial tumour, while the untreated tumour in the right lung was observed (**a**) The study flowchart is shown in (**b**) Pictures of rat, needle and syringe were adapted with permission from http://togotv.dbcls.jp/ja/togopic.2011.64.html and http://togotv.dbcls.jp/ja/togopic.2013.21.html published under CC BY 4.0 license.

The protocols for all experiments were approved by the Institutional Review Board (no. 17030). Furthermore, all procedures performed on animals complied with the Animal Research: Reporting of *In Vivo* Experiments guidelines. Overall, 160 male Sprague-Dawley rats, 4–5 weeks old and weighing 70–90 g, were purchased from SLC Inc. (Shizuoka, Japan) and housed individually with free access to food and water. General anaesthesia was induced before procedures via intramuscular injection of a 200 µl solution containing normal saline (180 µl), ketamine (800 µg, 16 µl) (Daiichi Sankyo Co., Tokyo, Japan), and xylazine (80 µg, 4 µl) (Bayer, Leverkusen, Germany). The flowchart of the present study is shown in Fig. 1.

**Figure 2:**
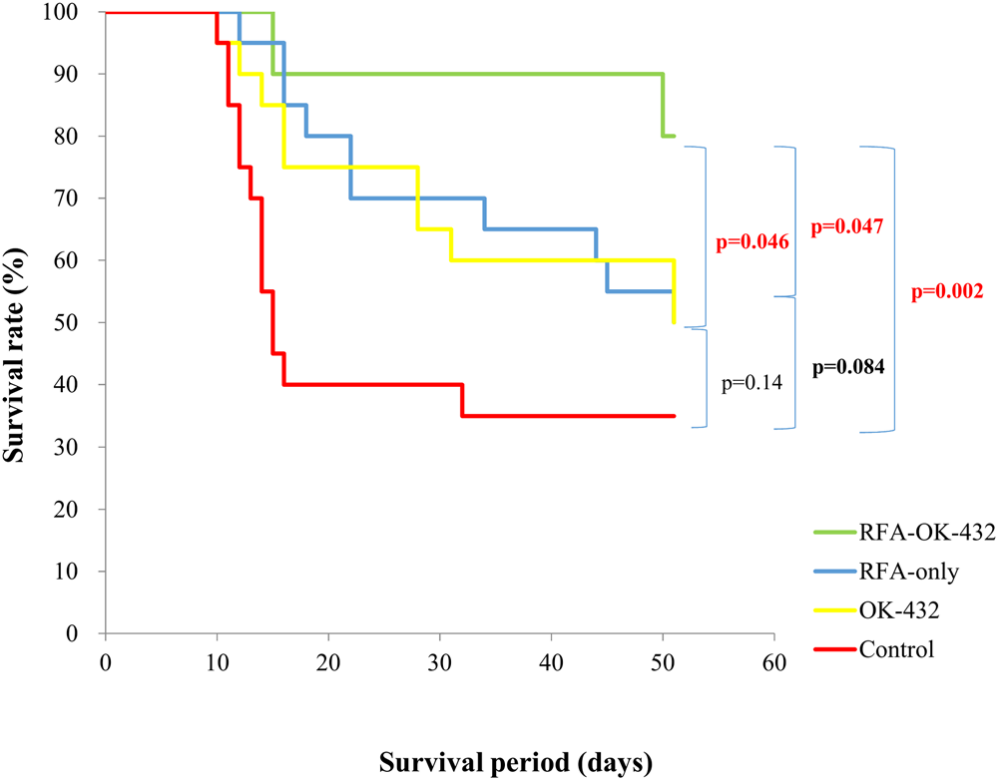
Kaplan-Meier survival curves are shown. The survival curves were compared using the log-rank test.

**Figure 3:**
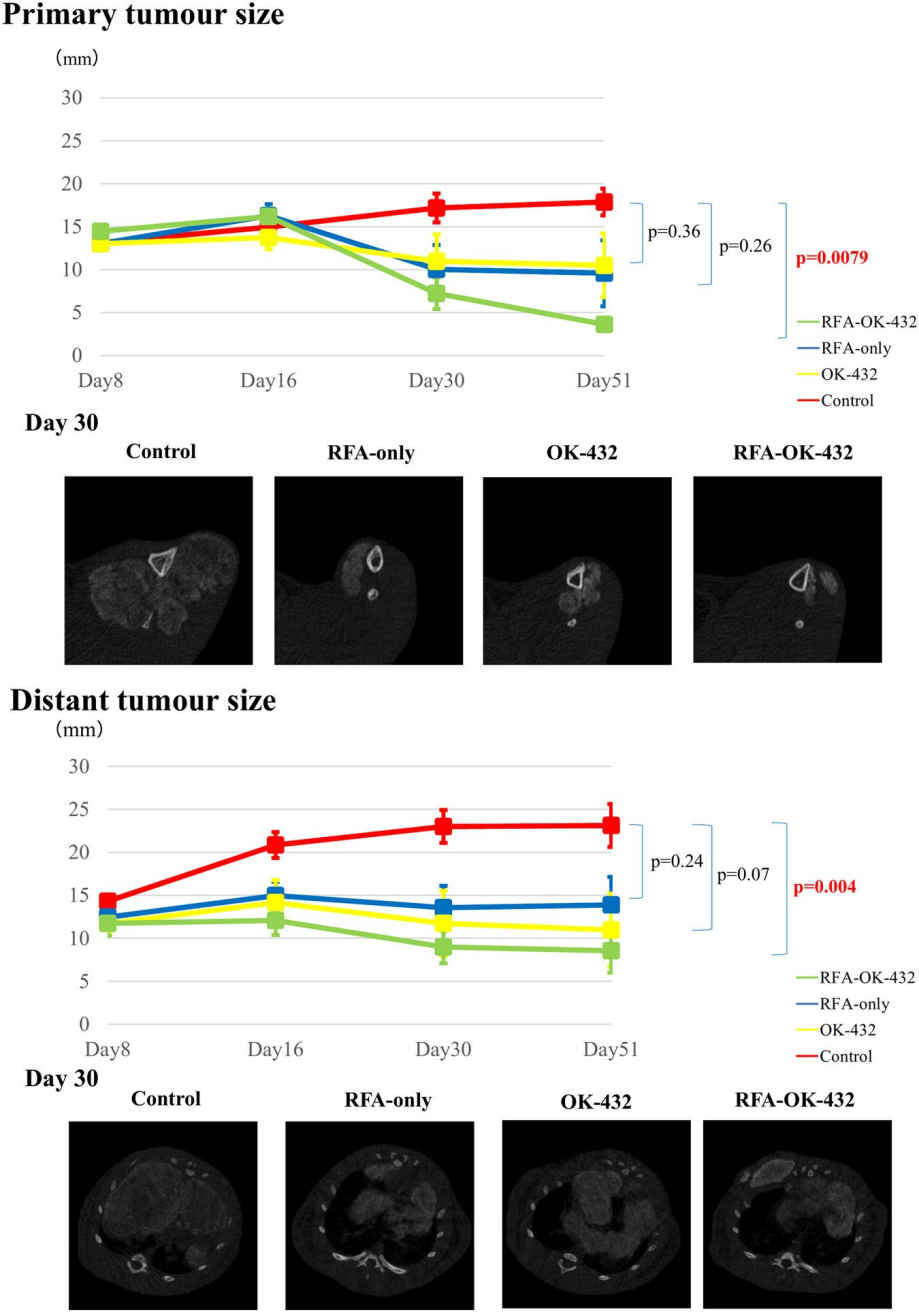
Maximum primary and distant tumour diameters were compared among the four groups: control (no treatment), RFA-only, OK-432 and RFA-OK-432 (RFA in combination with intratumoural OK-432 injection). The data are presented as the mean ± standard error. The figure shows the main axial images obtained via micro-computed tomography of the primary and distant tumour on day 30.

**Figure 4:**
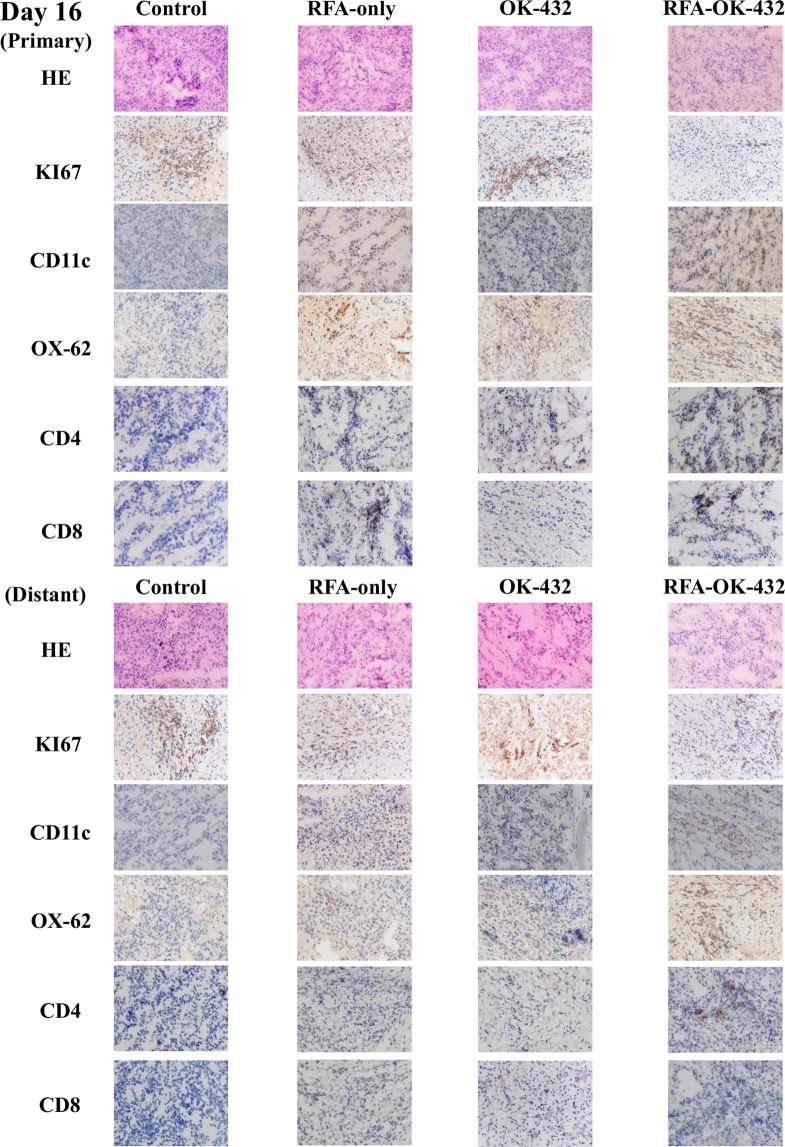
Haematoxylin-eosin examination findings in the primary and distant tumour obtained on day 16 are shown. Ki-67-positive cells, CD-11c+, OX-62+, CD4+, and CD8+ cells in the observed primary and distant tumour were detected via immunohistochemistry on day 16.

**Figure 5:**
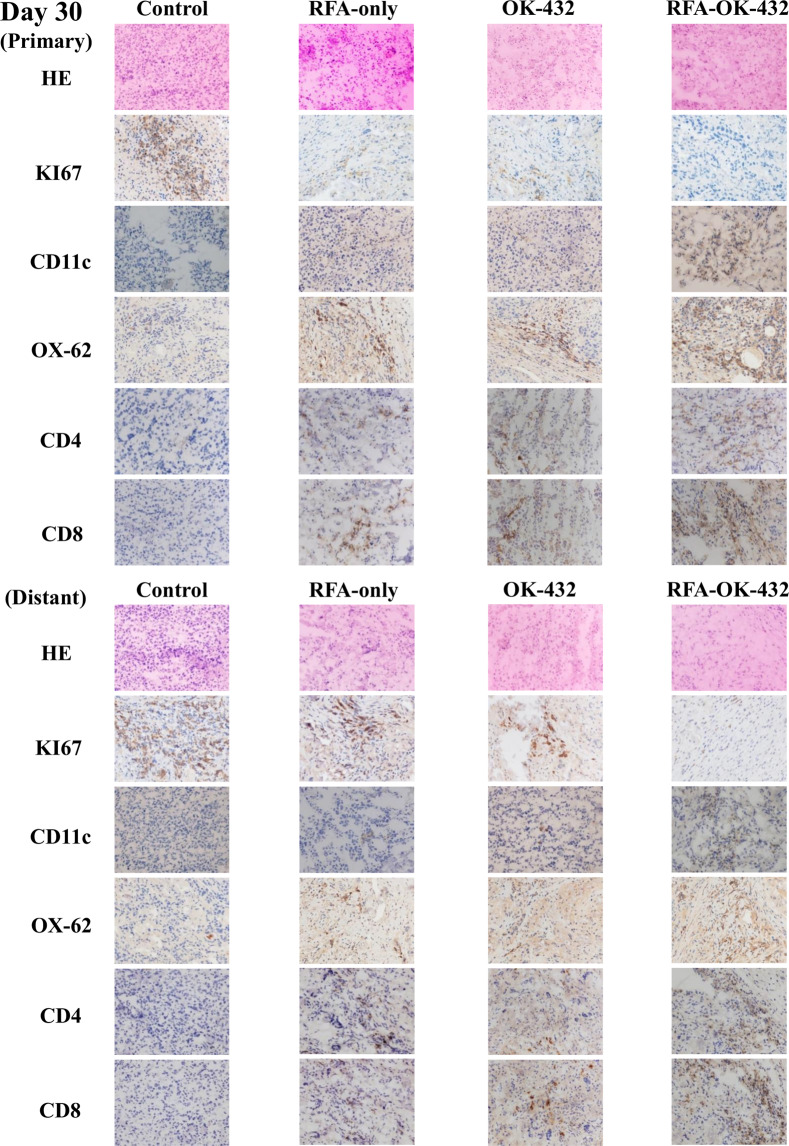
Haematoxylin-eosin examination findings in the primary and distant tumour obtained on day 30 are shown. Ki-67-positive cells, CD-11c+, OX-62+, CD4+, and CD8+ cells in the observed primary and distant tumour were detected via immunohistochemistry on day 30.

**Figure 6:**
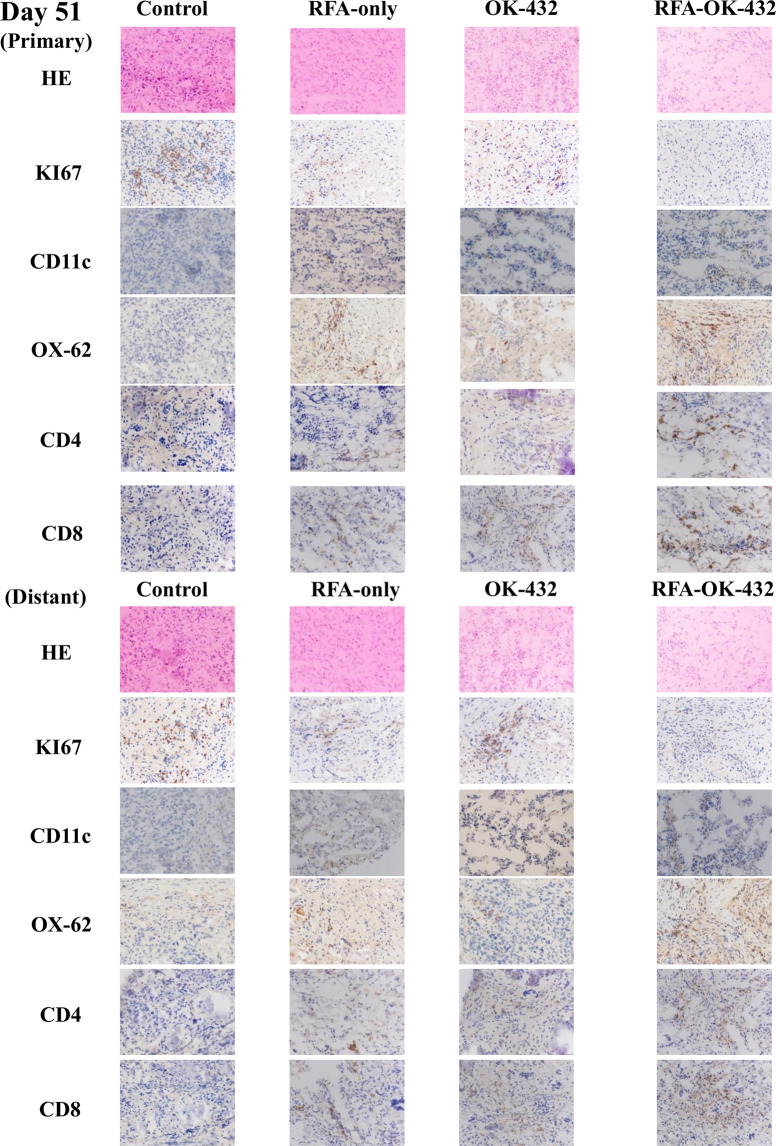
Haematoxylin-eosin examination findings in the primary and distant tumour obtained on day 51 are shown. Ki-67-positive cells, CD-11c+, OX-62+, CD4+, and CD8+ cells in the observed primary and distant tumour were detected via immunohistochemistry on day 51.

**Figure 7:**
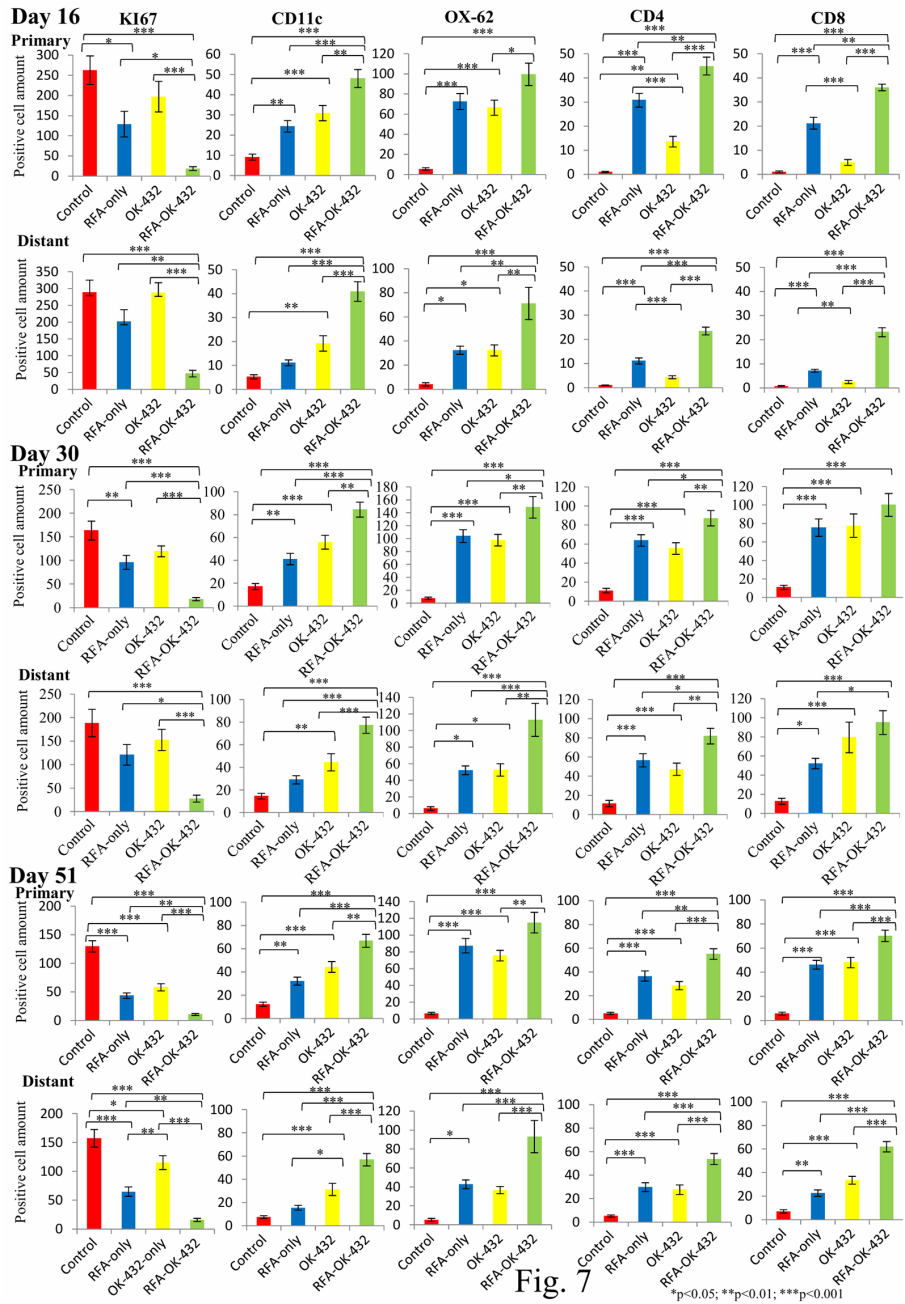
The number of positively-stained cells was counted with a microscope. This was achieved by selecting the largest number of cells among three chosen tumour section fields at ×200 magnification using Macro ImageJ. The data are presented as the mean ± standard error.

**Figure 8:**
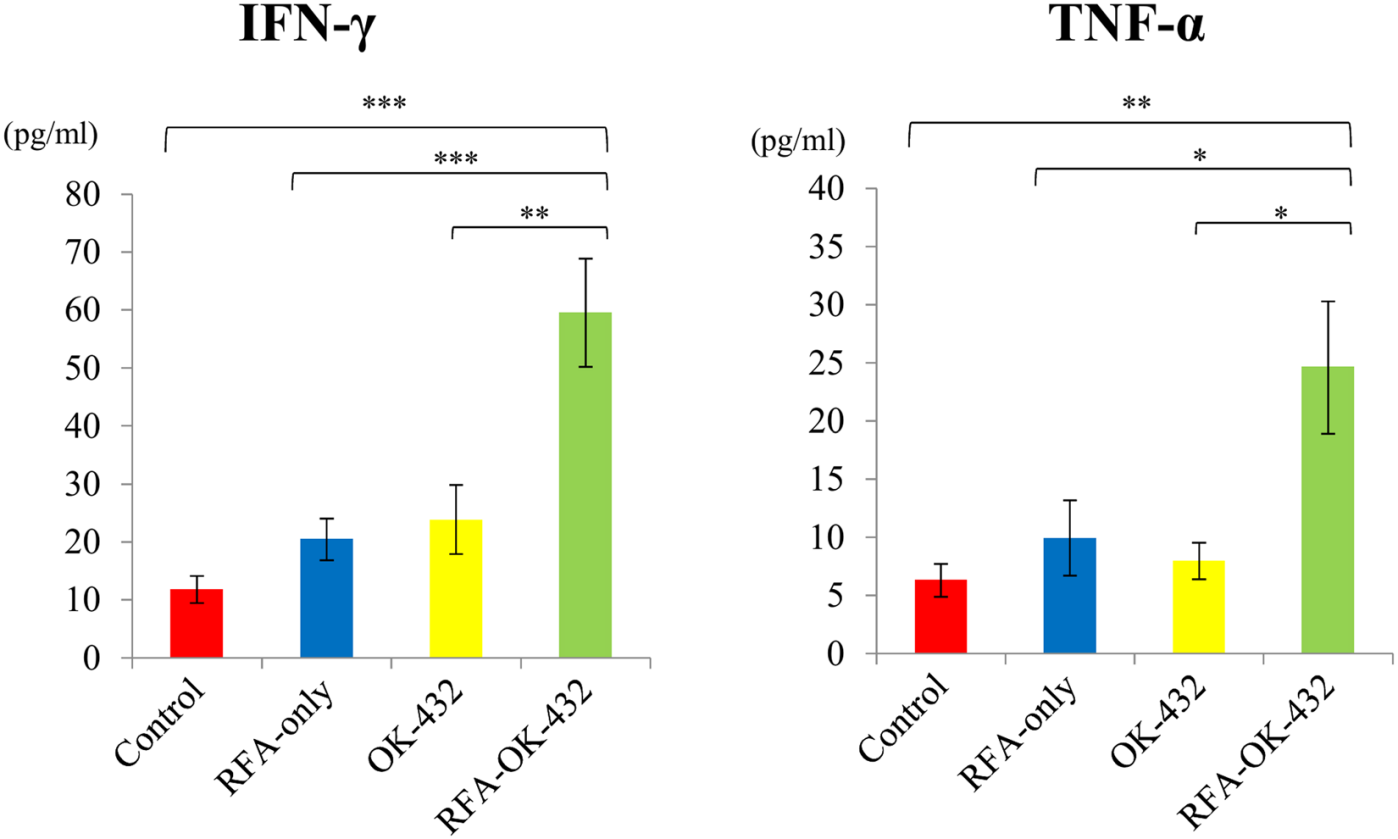
Cytokine level in plasma on day 30 was measured by ELISA.

### Experimental model

A rat osteosarcoma cell line (UMR106) was purchased from DS Pharma Promo Co., Ltd. (Osaka, Japan). These cells were maintained in a complete medium comprising high-glucose Dulbecco’s modified eagle medium formulated with 10% foetal bovine serum, 2 mM of L-glutamine, 100 μg/ml of streptomycin, and 100 units/ml of penicillin, cultured at 37 °C in 5% carbon dioxide, and confirmed to be mycoplasma-free.

Tumour implantation was performed by injecting 2 × 10^6^ UMR106 cells in 400 μl of 0.1 M phosphate-buffered saline fixed with 1.5% glutaraldehyde directly into the periosteum of both the left tibia and the right chest wall (day 0). The left tibial tumour was defined as the primary tumour and the right lung tumour as the distant metastatic tumour (Fig. 1).

Establishment of the implanted tumour was confirmed via micro-computed tomography (Latheta LCT-200; Hitachi-Aloka Medical, Tokyo, Japan) eight days after implantation. Rats with established tumours in both the left tibia and right lung on day 8 were used for further experiments. Tumour implantation yielded 145 rats with established tumours.

### RFA procedure

RFA was performed on day 9 using a LeVeen electrode (Boston Scientific, Natick, MA, USA) with an RF2000 generator (Boston Scientific) and eight retractable hooks with a maximum diameter of 2 cm. The electrode was transcutaneously inserted into the left tibial tumour, and retractable hooks were half-opened before ablation. After that, tumour ablation was performed at an output of 20 W and maintained until the generator automatically halted on reaching the maximum resistance because of increased impedance (e.g., roll-off).

### Intratumoural OK-432 injection

OK-432 powder containing a dose of 0.075 Klinische Einheit (KE) was dissolved in 150 µl of normal saline. Among rats in the RFA-OK-432 group, this solution was percutaneously injected into the ablation area of the left tibial tumour immediately after RFA on day 9.

### Study groups

Eighty of 145 rats were assigned to four groups for evaluation of the overall survival rate and tumour size: the control (n = 20, no treatment), RFA-only (n = 20, RFA to the left tibial tumour), OK-432 (n = 20, intratumoural OK-432 injection to the left tibial tumour), and RFA-OK-432 (n = 20, RFA with intratumoural OK-432 injection to the left tibial tumour) groups. The remaining 65 rats were also assigned to four groups for histological examination: the control (n = 20), RFA-only (n = 15), OK-432 (n = 15), and RFA-OK-432 (n = 15) groups. RFA and OK-432 were administered nine days after tumour implantation.

### Study endpoints

The survival duration was defined as the period between implantation and death with a final observation period of 51 days. The maximum diameters of both the left tibial tumours and right lung tumours were calculated on micro-computed tomography on days 8, 16, 30, and 51. At the end of the study, rats were euthanised with carbon dioxide.

### Histopathologic evaluation

Pathologic specimens of the remaining 65 rats were also prepared on days 8, 16, 30, and 51 after implantation: control (each n = 5 on days 8, 16, 30, and 51), RFA-only (each n = 5 on days 16, 30, and 51), OK-432 (each n = 5 on days 16, 30, and 51), and RFA-OK-432 (each n = 5 on days 16, 30, and 51). Histological examination was performed via haematoxylin-eosin and immunohistochemical staining of both the left tibial and right lung tumour specimens. Before immunohistochemical staining of both the left tibial and right lung tumour specimens, they were embedded in Sakura Tissue-Tek optimum cutting temperature compound (Sakura Finetek Japan Co. Ltd., Tokyo, Japan) for frozen tissue sections. These were labelled with primary antibodies (anti-CD11c; Abcam plc., Tokyo, Japan anti-CD4 and anti-CD8a; BD Biosciences, San Jose, CA, USA) for approximately 30 minutes. After that, they were labelled with a secondary antibody (N-Histofine Simple Stain Mouse Max PO [Gout] system; Nichirei Co., Tokyo, Japan) for approximately one hour. Further, tumour tissues were fixed for 24 h as 4-μm-thick paraffin-embedded sections. Sections of the tumour tissues were labelled with primary antibodies (Ki-67; 1000 dilution; Proteintech Group Inc., Tokyo, Japan anti-OX-62; 50 dilution; Santa Cruz Biotechnology Inc., CA, USA) at 4 °C overnight. Subsequently, they were also labelled with a secondary antibody for approximately one hour. Ki-67-positive tumour cells and tumour-infiltrating not only CD11c+ and OX-62+ cells but also CD4+ and CD8+ T cells were visualised using diaminobenzidine and haematoxylin and counter-stained.

Five tumours/groups were assessed. The number of positively-stained cells was counted via microscopy in three selected fields of tumour sections at ×200 magnification using ImageJ Macro (National Institutes of Health, USA)^14,15^. The greatest count from the chosen fields was selected, and the absolute cell number obtained on five slides per tumour was calculated. The absolute numbers of positively stained cells for every 25 spots/group were compared.

## ELISA

Blood (each n = 6: control, RFA-only, OK-432, and RFA-OK-432) was collected on day 30 via cardiac puncture into tubes containing sodium heparin. Plasma was obtained by centrifugation at 1000 × *g* for 10 min and stored at −90 °C until analysis. Concentrations of IFN-γ and TNF-α in plasma were measured by enzyme-linked immunosorbent assay (ELISA) using a High Sensitivity ELISA Kit (R & D Systems, Minneapolis, MN, USA).

### Statistical analyses

Statistical analyses were performed using Excel statistics software (version 2015; Social Survey Research Information Co. Ltd., Tokyo, Japan) for Windows.

Differences in survival among the four groups were evaluated using the Kaplan-Meier method and the log-rank test. The maximum diameters of the left tibial and right lung tumours were compared among the four groups via one-way analysis of variance (ANOVA) with the Tukey-Kramer multiple-comparison test. Temporal changes in the maximum diameters of right lung tumours in each group were compared via one-way ANOVA with the Bonferroni multiple-comparison test. Moreover, the number of Ki-67-positive tumour cells and that of tumour-infiltrating not only CD11c+ and OX-62+ cells but also CD4+ and CD8+ cells were compared among the four groups via one-way ANOVA using Tukey’s multiple-comparison test. The amount of IFN-γ and TNF-α in plasma were also compared among the four groups via one-way ANOVA using Tukey’s multiple-comparison test. *P* < 0.05 was considered statistically significant.

## Data Availability

The datasets generated and/or analysed during the current study are available from the corresponding author on reasonable request.
